# Richmond Academy of Medicine and Surgery

**Published:** 1898-01

**Authors:** Mark W. Peyser

**Affiliations:** Secretary and Reporter


### Society Reports.

### RICHMOND ACADEMY OF MEDICINE AND SURGERY.

Regular meeting held December 21, 1897. Dr. J. N. Upshur (President) in the chair. Dr. Mark W. Peyser, Secretary and Reporter.

Election of officers for the year 1898, resulted as follows: Dr. M. D. Hoge, Jr., President; Dr. E. C. Levy, First Vice-President; Dr. A. L. Wellford, Second Vice-President; Dr. A. C. Palmer, Third Vice-President; Dr. Mark W. Peyser, Secretary and Reporter; Dr. W. H. Parker, Assistant Secretary; Dr. R. B. Teusler, Treasurer; Dr. R. W. Nichols, Librarian.

### Reports of Cases.

*Vomiting of Blood by one-day-old Infant.*—The President reported a case of the like of which, he said, he had never seen before. On Sunday afternoon, 19th, he delivered a primapara, after an easy labor, of a girl baby weighing six pounds. It nursed heartily and there was no indication of trouble for twenty-four hours, when it threw up blood and colostrum. The history of the parents was unexceptional. Within a few hours after vomiting, there were frequent operations containing large amounts of meconium and blood. The skin and mucous membranes were pallid, the fontanelles depressed, and sutures prominent. He was at a loss to account for the hæmorrhage; but his idea was that there existed engorgement of the liver with congestion of the portal system and hyperæmia of the stomach and bowels. Thus, he prescribed calomel, two grains, and chalk, one grain, divided into ten powders, one powder given every hour. This afternoon (21st), the child was getting under the influence of the calomel, and as a result, there was less blood in the last three operations, and the child was progressing very satisfactorily. After mentioning the case to several medical friends who had never seen or heard of such a case, he consulted the *American Text-Book on Children*, and found reports of several cases with suggestion of just such treatment as he had given.

*Fulminant Appendicitis.*—Dr. George Ross said that in the past week he had had the only case of fulminating appendicitis he had ever seen. The patient was a man, age twenty-one, of feeble physique, night operator of the long-distance telephone. He had had bronchitis and recovered. On the night before his death, he seemed perfectly well. When seen at three P. M., there was a temperature of 102.5°; pulse, 130; and marked tenderness. At night, temperature was 101.5°, pain diminished, but general condition unfavorable. A consultation was held, with the result that the patient was taken to Virginia Hospital, but died before he could be placed on the table.

*Appendicitis—No Pathognomonic Symptom Usual.*—Dr. Ed. McGuire said he had seen a large number of cases of appendicitis, and had reached the conclusion that no single symptom could be relied upon to determine an operation. In the case of Dr. Neblett, there was suppurative peritonitis with a necrotic appendix. He had been improving, and on the evening of his death, was thought to be out of danger; but he was seized with convulsions, and in three hours was dead. The urine was loaded with albumen.

Last Monday morning, he saw a young man who had had a cramp the night before while on the train. There was a regular pulse and no fever. On the right side was a little tenderness. The bowels were free, but he had vomited. Thinking it was cholera morbus, calomel was prescribed. In the afternoon, upon making a second visit, was found increased tenderness, with a temperature of 100.8°. Appendicitis was diagnosed, and the patient taken to Virginia Hospital and operated upon the next day at one o'clock. A necrotic appendix was found. Recovery ensued.

Dr. McGuire said, in conclusion, that he had again and again seen cases where the symptoms were slight, but the course of the disease bad. He thought if all cases could be operated upon within twelve hours, mortality would be reduced one per cent.

*Occlusion of Opening Into Cæcum Possible Cause of Appendicular Abscess of Fulminant Appendicitis.*—Dr. W. T. Oppenhimer observed that in the fulminating form the appendix was always occluded next to the cæcum, distention resulting from accumulation of gas or pus. In a number of cases, the appendix could be cut off and no opening seen; therefore, he believed that in these the organ could be taken away close to the cæcum, no escape of pus resulting because of the agglutination. It was possible that a ligature was not necessary in these instances, and not applying one would prevent secondary hæmorrhage. He had observed cases without fecal fistula, in which the appendix had sloughed off and was floating in pus. Never having seen the point mentioned in any literature, he was of the opinion that it and the question of ligature should be investigated.

Dr. Hugh M. Taylor thought appendicitis capable of furnishing a greater number of surgical surprises than any disease with which he was familiar; and while it had claimed the lion's share of professional thought for the past ten years, we were only upon the threshold of knowledge as regards many of its most important phases. Its etiology was an open question; a terminal circulatory supply, a short mesentery, a structure of feeble resistance, an estuary in the fecal current often blocked, and an ever-present micro-organism were credited as etiological factors; but we must ascertain more of its causation before we could hope to do anything in the way of preventing it. Ideal surgery was, of course, preventive surgery, and it was to be hoped that future evolution of the subject might attain such an end. Individual and collective professional opinion as to appendicits—its etiology, symptoms and treatment—presented a succession of acrobatic changes. He thought he was correct in claiming that many more conversatives were becoming radical in their views as to the importance of early operative interference, and thorough work whenever the condition of the patient warranted. His experience fully sustained the conclusion that an early resort to operation found the patient sufficiently strong to endure complete work—i. e., strong enough to bear the prolonged anæsthesia, removal of the appendix and pus cavity, unmatting of the bowels, resection of infected omentum, etc. He would impress the idea that an early operation was conservative in that it sought to prevent pus collections and adhesions of the appendix, bowel and omentum, and the serious complication of septic, purulent or fibrino-plastic peritonitis. He contended that at some time in the history of every case of appendicitis, it was entirely a local phlegmon, and this was the elective period for operative interference.

Some of his friends, medical John Jaspers, rarely saw cases of appendicitis, and never met with cases calling for operation. When a practitioner of experience told him he never had torn perinei, he felt like telling him "Your eyesight is defective," or "You do not lift up the sheet to look." So when a man told him he never met with cases of appendicitis, he was tempted to urge him to study its symptoms, and was almost willing to promise him acquaintance with a surprising number of cases.

Typical appendicitis should be as easy to diagnose as typical pneumonia, typical typhoid fever, etc; but, unfortunately, we met with a good many cases which were atypical, and in this class, the differential diagnosis was not always easy. Three conditions in the right half of the abdomen and pelvis notably, presented symptoms simulating some one of the clinical types of appendicitis. He alluded to gall-tract and tubo-ovarian inflammations and displaced right kidney with renal or Dietle's crisis. The diagnosis was, of course, easier in men, inasmuch as cholelithiasis and its consequences—cholangitis, cholecystitis, empyema of the gall-bladder and gall-tract colic were so much more frequent in women. Displaced kidney and its effects were likewise more common in women, and usually occurred on the right side. Inflammation and suppuration from tubo-ovarian disease, and that incident to appendicitis, might present symptoms in common, but, usually, the differentiation could be made. The intimate lymphatic and vascular connection between the right broad ligament and the meso-appendix should be borne in mind, as it explained the frequent co-existence of appendicitis and right tubal inflammation. Conditions less frequently and less positively obscuring diagnosis of appendicitis were typhoid fever, ileus, intestinal indigestion, tubal gestation, gastric ulcer, hysteria, etc. He had at the present time, two cases of phantom appendicitis. In both, he was satisfied that the morbid condition existed only in the nervous system. Both patients were able to simulate many of the symptoms of appendicitis, as localized pain, muscular rigidity, etc. He was obliged to anæsthetize one in order to satisfy himself as to diagnosis, while the other spent sleepless nights and anxious days, and upon one occasion implored him to operate, so that he need not endure another such night of agony.

Dr. Ed. McGuire said if Dr. Oppenhimer's suggestions were followed, there would be liability to secondary infection. He had seen fecal matter in more than one instance in such cases.

